# α-Klotho expression determines nitric oxide synthesis in response to FGF-23 in human aortic endothelial cells

**DOI:** 10.1371/journal.pone.0176817

**Published:** 2017-05-02

**Authors:** Chih-Ping Chung, Yu-Chun Chang, Yan Ding, Kenneth Lim, Qinghua Liu, Langjing Zhu, Wei Zhang, Tzong-Shi Lu, Guerman Molostvov, Daniel Zehnder, Li-Li Hsiao

**Affiliations:** 1 Department of Neurology, Neurological Institute, Taipei Veterans general Hospital and National Yang Ming University, Taipei, Taiwan; 2 Department of Medicine, Renal Division, Brigham and Women’s Hospital, Harvard Medical School, Boston, United States of America; 3 Department of Nephrology, The First Affiliated Hospital, Sun Yat-sen University, Key Laboratory of Nephrology, Ministry of Health, Guangzhou, Guangdong, China; 4 Department of Nephrology, The Eighth Affiliated Hospital, Sun Yat-sen University, Shenzhen, Guangdong, China; 5 Department of Gastroenterology, Guizhou Cancer Hospital of Guizhou Medical University, Guiyang, Guizhou, China; 6 North Cumbria University Hospital NHS Trust, Carlisle, Cumbria, United Kingdom; Hungarian Academy of Sciences, HUNGARY

## Abstract

Endothelial cells (ECs) express fibroblast growth factor (FGF) receptors and are metabolically active after treatment with FGF-23. It is not known if this effect is α-Klotho independent or mediated by humoral or endogenous endothelial α-Klotho. In the present study, we aimed to characterize EC α-Klotho expression within the human vascular tree and to investigate the potential role of α-Klotho in determining FGF-23 mediated EC regulation. Human tissue and ECs from various organs were used for immunohistochemistry and Western blot. Primary cultures of human aortic endothelial cells (HAECs) and human brain microvascular endothelial cells (HBMECs) were used to generate *in vitro* cell models. We found endogenous α-Klotho expression in ECs from various organs except in microvascular ECs from human brain. Furthermore, FGF-23 stimulated endothelial nitric oxide synthase (eNOS) expression, nitric oxide (NO) production, and cell proliferation in HAECs. Interestingly, these effects were not observed in our HBMEC model *in vitro*. High phosphate treatment and endothelial α-Klotho knockdown mitigated FGF-23 mediated eNOS induction, NO production, and cell proliferation in HAECs. Rescue treatment with soluble α-Klotho did not reverse endothelial FGF-23 resistance caused by reduced or absent α-Klotho expression in HAECs. These novel observations provide evidence for differential α-Klotho functional expression in the human endothelium and its presence may play a role in determining the response to FGF-23 in the vascular tree. α-Klotho was not detected in cerebral microvascular ECs and its absence may render these cells nonresponsive to FGF-23.

## Introduction

Normal vascular function is critically dependent on physiological endothelial cell (EC) responses to altered tissue nutrient demand and an appropriate stress response. The endothelium generates many factors that regulate vascular tone, adhesion of circulating blood cells, smooth muscle proliferation, and inflammation; as such, EC dysfunction has emerged as a relevant risk marker for cardiovascular events in the general population, particularly older individuals [1], patients with diabetes [2], or renal impairment [3]. Recently the phosphate and vitamin D regulating hormone fibroblast growth factor 23 (FGF-23) has also been implicated in cardiovascular risk of patients with normal and impaired renal function [4, 5]. FGF-23 exerts these cellular effects through the fibroblast growth factor receptor (FGFR) family/α-Klotho cell surface receptor complex [6]. α-Klotho may form this receptor complex with FGFR1 (120 kDa), FGFR3 (97 kDa), and FGFR4 (87 kDa) [7]. First published in 1997, α-Klotho is a type I single-pass transmembrane full-length protein (130 kDa) that may generate an additional soluble form through proteolytic cleavage [8]. The likely major source for circulating, soluble α-Klotho is the kidney. We have recently shown that transmembrane cell surface α-Klotho is expressed in numerous human tissues beyond the kidney and parathyroid gland, including the smooth muscle cell layer of the artery wall [9, 10].

Both FGF-23-deficient and α-Klotho deficient animal models exhibit similar phenotypes as patients with impaired renal function, e.g., accelerated aging with the development of extensive arteriosclerosis and vascular calcification [4, 11]. A nephron specific α-Klotho knockout mouse model showed similar phenotypes to the systemic α-Klotho knockout mouse including premature aging and arteriosclerosis [10]. Similarly, other studies with α-Klotho knockout mice display attenuation of endothelium dependent vascular protection along with decreased aortic and arteriolar vasodilation as a result of reduced nitric oxide (NO) production [12, 13]. Rescue treatment with *in vivo* α-Klotho delivery resulted in reversal of endothelial dysfunction, reduction of the elevated blood pressure, and prevention of arterial wall hypertrophy and perivascular fibrosis [13]. Interestingly, some suggest that high circulating concentrations of FGF-23 impaired endothelium dependent vascular relaxation by increasing superoxide levels and reducing NO bioavailability [14]. Furthermore, soluble α-Klotho mitigated FGF-23 or high phosphate mediated vasoconstriction and reduced NO production in human umbilical vein endothelial cells (HUVECs) [15]. Important questions to address are whether full-length transmembrane α-Klotho is expressed in ECs of human artery wall and whether α-Klotho is important for endothelium function.

In this study, we present data that show differential expression of functional full-length α-Klotho protein in human ECs. In addition, the study provides evidence that this full-length α-Klotho protein is required for EC response to FGF-23.

## Material and methods

### Tissue samples

This study conforms to the principles outlined in the Declaration of Helsinki. Human tissues were obtained with local ethical approval and informed written consent from three sources: surgical specimens from the human tissue bank at University Hospitals Coventry and Warwickshire NHS Trust, UK (Ethics approval 130072), artery and kidney tissue from Warwick Medical School, UK (Ethics approval 05/Q2802/26 and 10/H12111/36), and brain tissue was kindly donated by the New York Brain Bank (NYBB) at Columbia University.

### Cell culture and cell lysates

Three different age-matched sources of human aortic endothelial cells (HAECs) (Cat No. 6100; ScienCell Research Laboratory, Carlsbad, CA) and two different age-matched sources of human brain microvascular endothelial cells (HBMECs) (Cat No. ACBRI376; Cell Systems, Kirkland, WA) were obtained. The number (*n*) corresponds to the total repeat experiments performed collectively by using all 2 or 3 cell sources. Cells were treated with EC medium (ECM, Cat No. 1001; ScienCell Research Laboratory, Carlsbad, CA) containing 5% FBS and 0.5 mM β-glycerolphosphate for HAECs, and CSC medium (Cat No. 4Z3-500; Cell Systems, Kirkland, WA) containing 5% FBS for HBMECs. FGF-23 (Cat No. 2604-FG; R&D Systems, Minneapolis, MN) was used for cell treatments. Passages of cells used in this study were less than or equal to eight. HBMECs were assessed regularly to confirm their central nervous system properties. The cells were stained with antibodies for Von Willebrand factor to confirm EC origin. These cultures were analyzed routinely for astrocyte contamination by staining with anti-glial fibrillary acidic protein. Cell lysates of human pulmonary microvascular endothelial cells (HPMECs, Cat No.3006) and human cardiac microvascular endothelial cells (HCMECs, Cat No. 6006) for Western blot were commercially obtained (ScienCell Research Laboratory, Carlsbad, CA).

### Immunohistochemistry

Antigen retrieval of formalin fixed and paraffin-embedded human tissue sections were achieved using a pressure cooker. Rabbit polyclonal α-Klotho antibody (Cat No. Ab69208; Abcam, Cambridge, MA) and rabbit polyclonal isotype antibody (Cat No. ab27478) at 1:100 to 1:500 were used. Sections were stained with Vectastain Universal Elite ABC (Avidin and Biotinylated horseradish peroxidase macromolecular Complex solution) kit (Cat No. PK-6200; Vector Laboratories, Burlingame, CA) before counterstaining with hematoxylin.

### Cellular proliferation assay

Cell proliferation was assessed using XTT *in vitro* assay kit (Cat No. TOX2; Sigma-Aldrich, Louis, MO). Briefly, cells were seeded until 50% confluent in complete medium. FGF-23 treatment was then performed in DMEM containing 0.5% FBS. XTT stock solution equal to 20% of the culture medium volume was added to media. Media containing XTT was then transferred to a 96-well plate and the absorbance was measured at 450 nm. Background absorbance readings at 690 nm were subtracted from readings taken at 450 nm.

### NO assay

NO levels were assessed with a Colorimetric Nitric Oxide assay kit (Cat No. 8098; ScienCell Research Laboratory, Carlsbad, CA) according to the manufacturer’s instructions. The unstable nature of NO means its rapid oxidative degradation to nitrite and nitrate. Subsequently the nitrate was reduced to nitrite with vanadium (III) chloride and then the total nitrite was quantified by Griess reaction. The tested samples were fresh cell lysate after cell treatment experiments. Cells were washed with cold PBS, resuspended in ice-cold Assay Buffer, and homogenized quickly on ice. Then the samples were centrifuged for 5 minutes at 4°C at top speed to remove any insoluble material. The collected supernatant underwent a deproteinization step before being used in the assay. After these reactions, the absorbance was measured with a test wavelength at 540 nm and a reference wavelength at 630 nm. NO production for each sample was normalized by protein concentration.

### Western blot

Western blot technique was used for protein analysis of cell lysates as previously described [12]. Briefly, sample media was mixed with 4X loading (sample) buffer (Sigma, St Louis, MO) and Radio-Immuno Precipitation Assay buffer, pH 7.4 (Cat No. BP-115, Boston BioProducts, Ashland, MA). 15–50 μg of sample was subjected to Western blot depending on target proteins. The same amount of protein was used within a Western blot and target proteins were then visualized with an enhanced chemiluminescence detection system. Antibodies used in this study were for endothelial nitric oxide synthase (eNOS) (Cat No. 9572; Cell signaling technology, Danvers, MA) 1:1000; phospho-eNOS (Ser1177) (p-eNOS) (Cat No. 9571; Cell signaling technology, Danvers, MA) 1:1000; α-Klotho (Cat No. Ab75023; Abcam, Cambridge, MA) 1:1000; phospho-FGFR (Tyr653/654) (p-FGFR) (Cat No.3471; Cell signaling technology, Danvers, MA) 1:1000; FGFR1 (Cat No. SC-121; Santa Cruz Biotechnology, Santa Cruz, CA) 1:1000; FGFR3 (Cat No. SC-123; Santa Cruz Biotechnology, Santa Cruz, CA) 1:1000; FGFR4 (Cat No. Ab5481; Abcam, Cambridge, MA) 1:1000 and Actin (Cat No. MAB1501; Millipore, Billerica, MA) 1:1000.

### α-Klotho siRNA transfection

α-Klotho small interfering RNA (siRNA) was purchased from Invitrogen (Cat No. 4392422; Carlsbad, CA). Cells were seeded until 60% confluent and placed in opti-MEM I Reduced Serum Medium (Cat No. 31985–062; Invitrogen, Carlsbad, CA). Transfection was achieved using Lipofectamine reagent (Cat No. 15338–100) and PLUS reagent (Cat No. 11514–015) from Invitrogen (Carlsbad, CA), at the manufacturer’s recommended concentrations. For all experiments, 1 μM α-Klotho siRNA was used over a transfection time of 24 hours followed by EC medium and the other cell treatments. Validation of siRNA efficacy experiments was performed (S1 Fig).

### Reverse transcription-polymerase chain reaction (RT-PCR)

Total RNA was isolated from cell lysates using an RNeasy kit (Qiagen,Hilden, Germany) following the manufacturer's protocol. Reverse transcription of total RNA (100–200 ng) was carried out using Superscript 3 reverse transcriptase (Invitrogen, Carlsbad, CA) with random hexamers (Bioline, Taunton, MA). The generated cDNA was used as the template for PCR of α-Klotho using BIOTAQ DNA polymerase (Bioline, Taunton, MA). The primers for α-Klotho were: forward, 5’- ACT CCC CCA GTC AGG TGG CGG TA-3’ and reverse, 5’- TGG GCC CGG GAA ACC ATT GCT GTC-3’ [16, 17]. PCR products were then loaded for agarose gel electrophoresis.

### Statistics

Data were shown as mean ± SD. The 2-tailed Student’s test was used to compare data between two experimental groups. ANOVA with Bonferroni *post-hoc* analysis was performed to compare data among experimental groups if there were more than 2 groups.

## Results

### FGF-23 increases cell proliferation, NO production, eNOS protein expression, and eNOS activation in human aortic but not brain microvascular endothelial cells, *in vitro*

**Fig 1:**
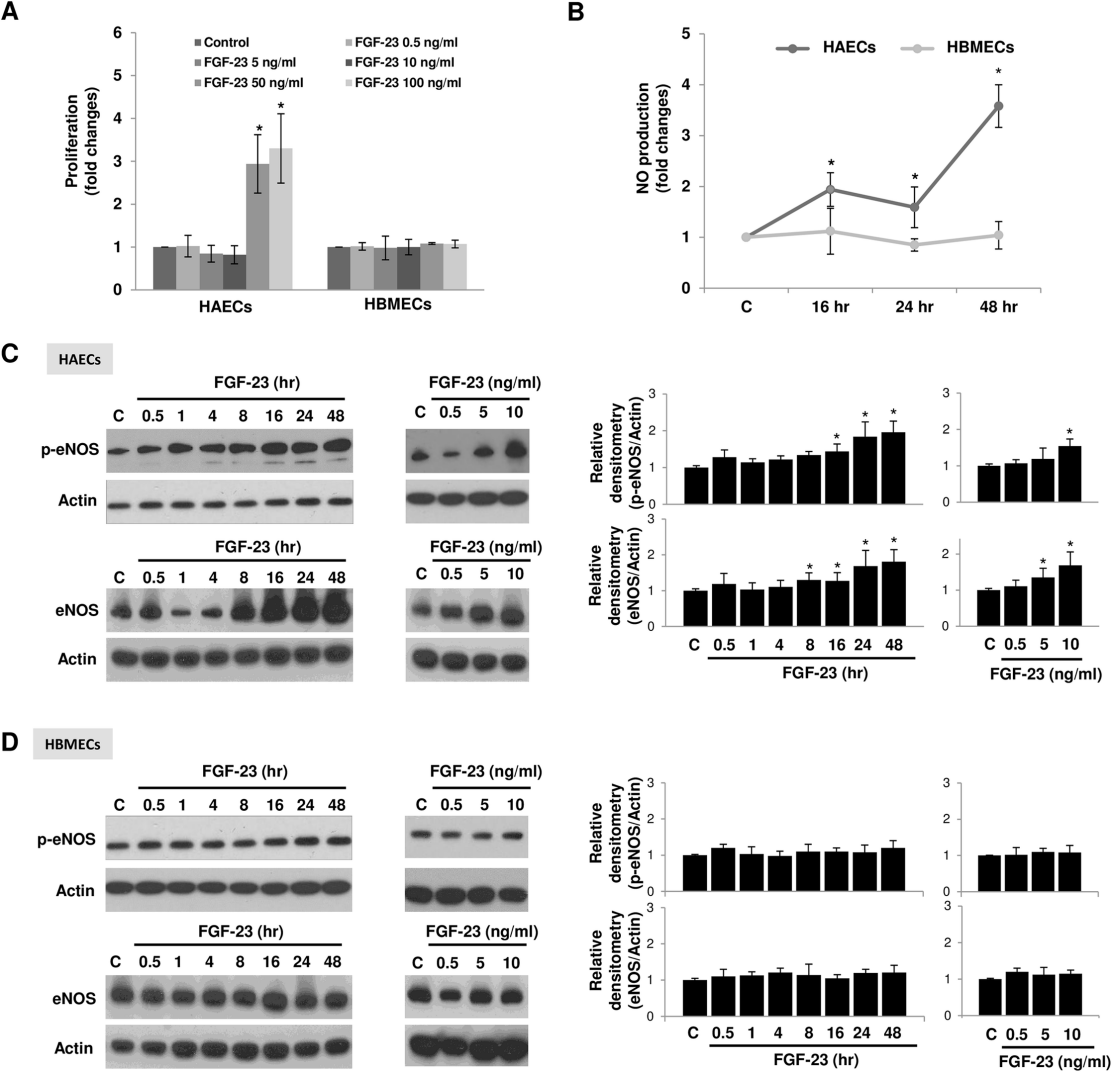
FGF-23 increases cell proliferation, NO production, and eNOS protein expression and activation in human aortic endothelial cells (HAECs) but not in human brain microvascular endothelial cells (HBMECs). **(A)** FGF-23 treatment for 48 hours stimulates proliferation in HAECs at 50 and 100 ng/ml, but not in HBMECs; *n = 6*. **(B)** NO production of HAECs was increased following treatment with 10 ng/ml FGF-23 for 16, 24, and 48 hours compared with controls. There was no FGF-23-stimulated NO production in HBMECs; *n* = 6. **(C)** FGF-23 increased phospho-eNOS (Ser1177) and eNOS protein expression in a time- and dose-dependent manner in HAECs. The phospho-eNOS (Ser1177) and eNOS protein expression of HAECs following stimulation with 10 ng/ml of FGF-23 for indicated periods of time and after incubation with FGF-23 at indicated concentrations for 48 hours was detected by Western blot; *n* = 6. **(D)** Time- and dose-response of FGF-23 on HBMECs, displayed no effect on phospho-eNOS (Ser1177) and eNOS protein expression; *n* = 6. Quantitative analysis of Western blot by densitometry is normalized to actin. Data represent mean ± SD of at least 3 independent experiments. *Significantly different versus control by ANOVA test with Bonferroni *post-hoc* analysis.

We first investigated whether FGF-23 stimulates EC proliferation as observed in kidney cells [18] and vascular smooth muscle cells (SMCs) [12]. Our results showed that FGF-23 stimulated proliferation of HAECs, *in vitro* only when cells were treated with FGF-23 concentrations > 50 ng/ml. FGF-23 had no effects on the proliferation of HBMECs (Fig 1A).

We next investigated whether FGF-23 could play a role in regulating endothelial NO production in human ECs. The results showed that NO production was increased in HAECs (Fig 1B). These effects were observed using an FGF-23 concentration of 10 ng/ml, a biological response dose, and consistent with previous published studies [12]. Interestingly, FGF-23 had no such effects in HBMECs (Fig 1B). In addition, dose-response and time-dependent studies with HAECs showed that FGF-23 increased not only eNOS expression, but also eNOS phosphorylation at serine 1177 (a site associated with eNOS activation), from 8 hours and 16 hours, respectively (Fig 1C). Once again, FGF-23 had no effects on phosphorylated eNOS and eNOS expressions in HBMECs (Fig 1D).

### Endogenous α-Klotho is expressed in endothelial cells, *in vivo* and *in vitro*, except in the central nervous system’s microvasculature

**Fig 2:**
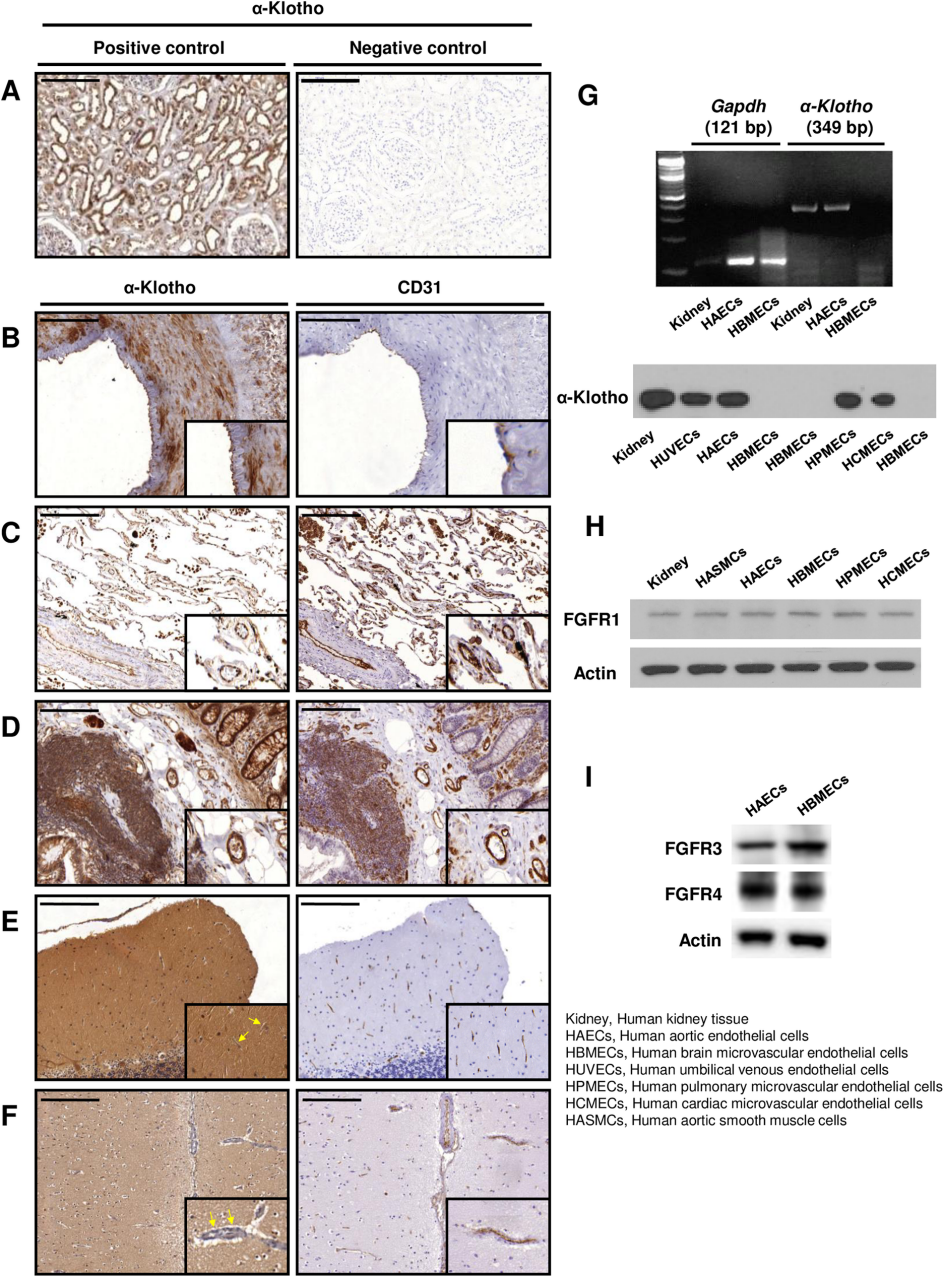
Full-length α-Klotho expression profiles in human endothelial cells, *in vivo* and *in vitro*. **(A)** With immunohistochemistry, kidney tissues were used with anti-Klotho antibody as a positive control and with isotype antibody as a negative control. **(B-F)** Using immunohistochemical analysis of endothelium in various human tissues showed that α-Klotho protein was expressed in renal artery endothelium (B), in lung microvessels (C), and in colon submucosa (D). α-Klotho expression was not shown in microvascular ECs from cerebellum (E) and hippocampus (F) regions. Endothelium specific marker CD31 was used to stain consecutive tissue sections. Yellow arrows point to microvascular ECs. *n* = 5 in tissues of each site; Scale bar = 200 μm. **(G)** *In vitro* studies showed that the α-Klotho gene expression (upper panel) was detected in human aortic endothelial cells (HAECs), but not in human brain microvascular endothelial cells (HBMECs) with kidney as a positive control; *n* = 6. Western blot (lower panel) confirmed full-length α-Klotho protein expression in human umbilical venous endothelial cells (HUVECs), HAECs, pulmonary microvascular endothelial cells (HPMECs), and cardiac microvascular endothelial cells (HCMECs). No α-Klotho protein expression was found in HBMECs; *n* = 3. **(H)** Fibroblast growth factor receptor 1 (FGFR1) protein was expressed widely in human endothelial cells originating from various human tissues *in vitro*; n = 3. **(I)** Fibroblast growth factor receptor 3 (FGFR3) and fibroblast growth factor receptor 4 (FGFR4) were expressed in both HAECs and HBMECs; *n* = 3.

We have recently shown tissue-wide distribution for locally synthesized, full-length membrane α-Klotho with the use of a targeted proteomics approach. Expression was described in epithelial, endocrine, and neuronal cells as well as cells of the artery wall [13]. Here we first confirmed expression of α-Klotho in tubular epithelial cells of kidney by immunohistochemistry, as a positive control (Fig 2A). We then investigated the expression profile of EC α-Klotho in the vascular tree that, to the best of our knowledge, no published studies to-date has yet explored. CD31 is an endothelial-specific marker and, as expected, was identified across the endothelial monolayers in this study. α-Klotho protein expression was detected in ECs from renal arteries (Fig 2B), pulmonary microvessels (Fig 2C), and colon submucosa microvessels (Fig 2D). Brain tissues from the cerebellum and hippocampus regions were used to investigate the expression of α-Klotho in brain microvessels. While we found α-Klotho expression in neuronal cells of the brain tissues, consistent with our previous findings [13], its expression was not detectable in microvessel ECs of cerebellum (Fig 2E) and hippocampus (Fig 2F). Furthermore, using primary ECs, α-Klotho gene expression was found in HAECs but not in HBMECs (Fig 2G, upper panel). With Western blot, the full-length α-Klotho (130 kDa) protein was found in HUVECs, HAECs, HPMECs, and HCMECs, but not in HBMECs (Fig 2G, lower panel). Our results suggest that the α-Klotho expression in human ECs is tissue specific and not uniform among the various tissue-types.

To investigate if the FGFR/α-Klotho complex mediates ECs response to FGF-23, we then detected FGFR expression. FGFR1 was expressed in all ECs analyzed, including HAECs, HPMECs, HCMECs, and HBMECs (Fig 2H). Furthermore, FGFR3 and FGFR4 expression was also observed in both HAECs and HBMECs (Fig 2I). The altered FGF-23 responses between HAECs and HBMECs coupled with differential endothelial α-Klotho protein expression along the human vascular tree suggest that the EC response to FGF-23 might be primarily dependent on α-Klotho.

### FGF-23-induced NO production and eNOS up-regulation are mitigated under high phosphate condition, a state of α-Klotho deficiency

**Fig 3:**
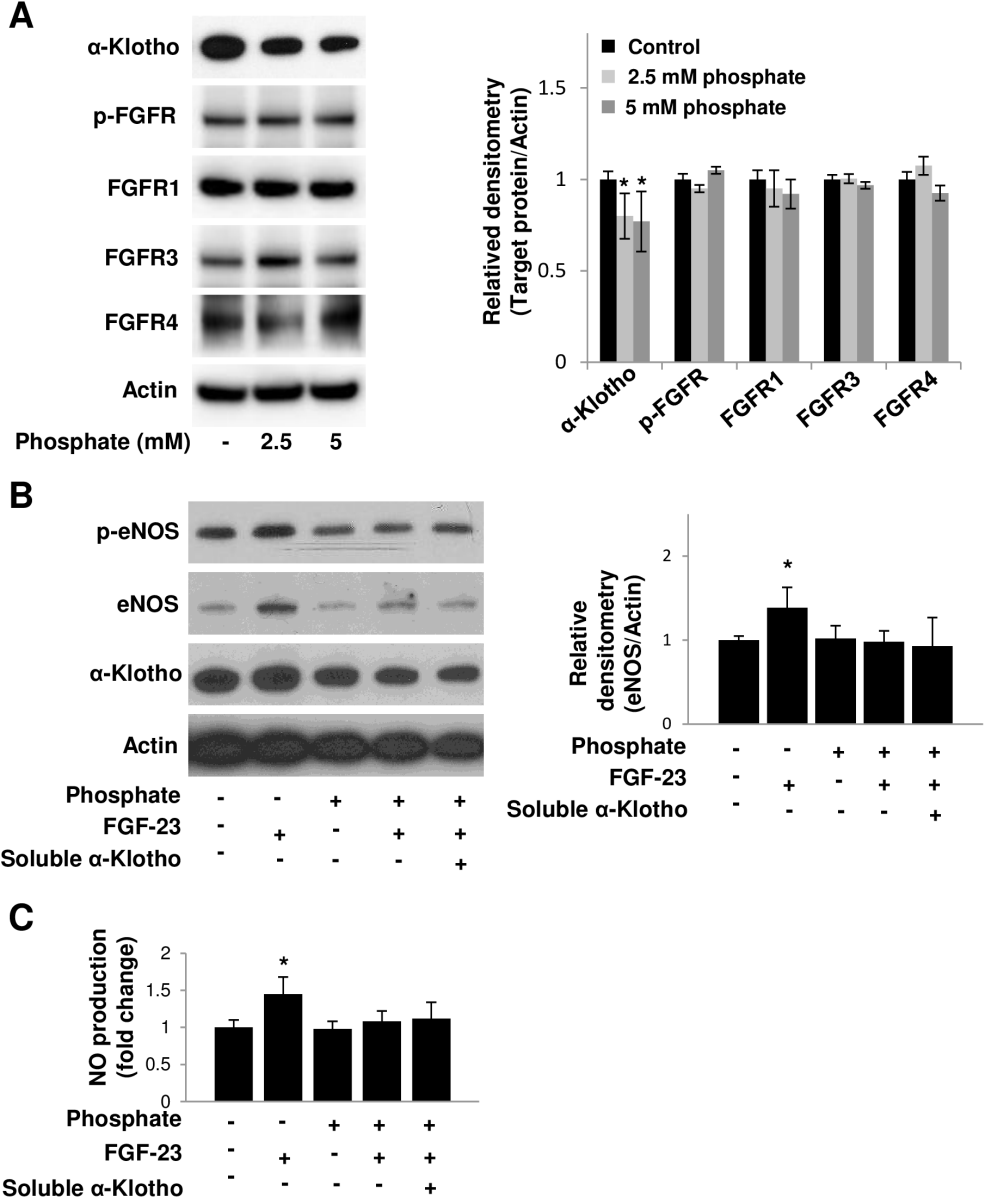
FGF-23-induced NO production and eNOS up-regulation are mitigated under high phosphate treatment, a state of α-Klotho deficiency. **(A)** Endogenous α-Klotho expression in HAECs was suppressed after high phosphate treatment (2.5 and 5 mM β-glycerophosphate) for 24 hours. Phosphate did not influence phospho-FGFR (Tyr653/654), FGFR1, FGFR3, and FGFR4 expression; *n* = 6. **(B and C)** High phosphate (5 mM β-glycerophosphate) treatment mitigated FGF-23’s effect (10 ng/ml) on increased protein expression of phospho-eNOS (Ser1177) and eNOS (B) and NO production (C) in HAECs; *n* = 6. Soluble α-Klotho (0.2 nM; 26 ng/ml) did not render high phosphate-treated HAECs responsive to FGF-23. Quantitative analysis of Western blot by densitometry is normalized to actin. Data represent mean ± SD of at least 3 independent experiments. *Significantly different versus control by ANOVA test with Bonferroni *post-hoc* analysis.

We have previously shown that exposure of human aortic SMCs to high phosphate renders them FGF-23 resistant due to loss of full-length α-Klotho expression [12]. When HAECs were treated under high phosphate conditions of 2.5 mM and 5 mM [12], α-Klotho protein expression was significantly suppressed. On the other hand, p-FGFR (Tyr653/654), FGFR1, FGFR, and FGFR4 protein expression of HAECs was not altered after exposure to high phosphate (Fig 3A). The FGF-23 mediated up-regulation of p-eNOS and eNOS expression (Fig 3B) and increased NO production (Fig 3C) were also blocked after high phosphate treatment; adding soluble α-Klotho to HAECs treated with high phosphate could not rescue EC resistance to FGF-23 (Fig 3B and 3C). Soluble α-Klotho concentration in human circulation has been reported to range from 0.061 to 0.74 ng/ml [19]; in this study, we used concentrations of 0.2 X 10^−3^, 0.2 X 10^−2^, 0.2 X 10^−1^ and 0.2 nM (0.026–26 ng/ml).

### FGF-23-induced cell proliferation, NO production, and eNOS up-regulation in endothelial cells are mediated by endogenous α-Klotho

**Fig 4:**
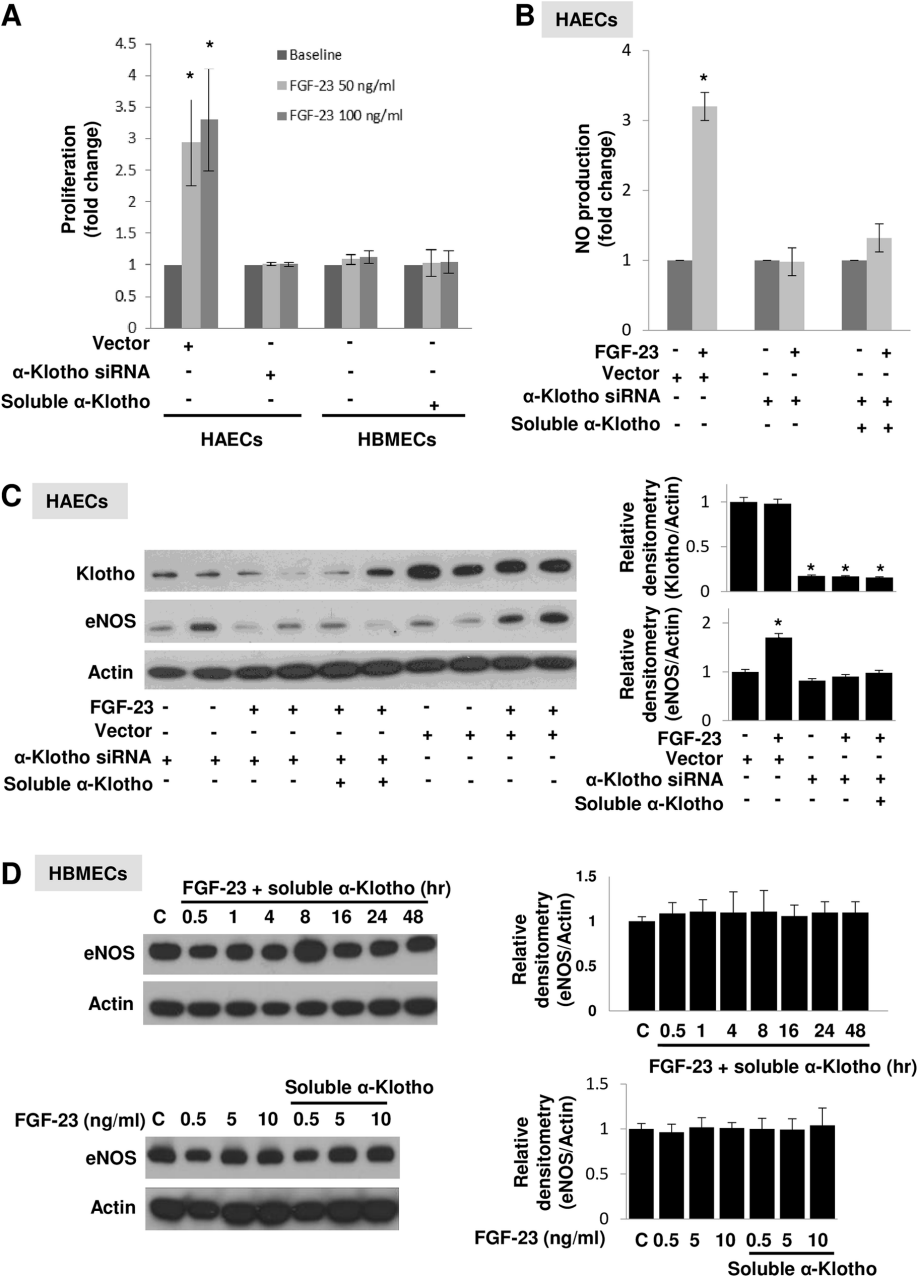
Endothelial FGF-23 response depends on full-length α-Klotho but not soluble α-Klotho. **(A)** FGF-23 treatment mediated proliferative effect on HAECs was mitigated by α-Klotho siRNA treatment. Treating with soluble α-Klotho (0.2 nM, 26 ng/ml) did not render HBMECs responsive to FGF-23; *n* = 3. **(B)** FGF-23 mediated NO production in HAECs was mitigated by α-Klotho siRNA treatment. Treating with soluble α-Klotho (0.2 nM, 26 ng/ml) did not render HAECs with α-Klotho knockdown responsive to FGF-23; *n* = 3. **(C)** HAEC α-Klotho knockdown with α-Klotho siRNA abrogated FGF-23 mediated eNOS protein up-regulation. Scrambled siRNA served as control (Vector) and display maintained endothelial FGF-23 response. Addition of soluble α-Klotho could not rescue HAEC resistance to FGF-23; *n* = 3. **(D)** HBMECs remained resistant to FGF-23 treatment with no increased expression of eNOS protein, time- and dose-dependent studies; *n* = 3. Quantitative analysis of Western blot by densitometry is normalized to actin. Data represent mean ± SD of at least 3 independent experiments. *Significantly different versus control by ANOVA test with Bonferroni *post-hoc* analysis.

Our group and others previously reported that FGF-23-Klotho signaling stimulates proliferation in vascular SMCs [12] and renal epithelial cells [18]. To confirm the dependence of EC response to FGF-23 on full-length and not circulating α-Klotho, knockdown experiments in HAECs were performed. The FGF-23 mediated increases in proliferation, NO production, and expression of eNOS in HAECs were abrogated after α-Klotho knockdown (Fig 4A, 4B and 4C). Importantly, adding soluble α-Klotho to HAECs following α-Klotho knockdown was not able to rescue EC resistance to FGF-23 (Fig 4B and 4C). This suggests that ECs are only responsive to FGF-23 in the presence of full-length α-Klotho synthesized in ECs.

Regardless of the presence of the FGFR1, FGFR3, and FGFR4 (well-known receptors for FGF-23) [6, 7], HBMECs *in vitro* did not respond to FGF-23 due to the absence of full-length α-Klotho. We therefore investigated if circulating α-Klotho made HBMECs responsive to FGF-23. After adding soluble α-Klotho, HBMECs remained resistant to FGF-23 and no increased cellular proliferation or expression of eNOS was seen (Fig 4A and 4D).

## Discussion

In this study we showed for the first time full-length α-Klotho protein expression in the ECs of elastic arteries, medium sized arteries, and in the microcirculation of numerous human tissues, and described differential endothelial α-Klotho protein expression along the human vascular tree. ECs express functionally active α-Klotho protein in most vascular beds; however, no α-Klotho protein was found in the microcirculation of brain or brain tissue derived ECs. We reveal that FGF-23 mediated eNOS protein expression, NO production, and cell proliferation in ECs with the expression of membrane α-Klotho protein. On the other hand, there was a lack of functional response of brain derived ECs to FGF-23, regardless of the presence of the endothelial FGF receptors or soluble α-Klotho. Our results suggest that FGF-23-mediated endothelial functions require the presence of full-length membrane α-Klotho protein.

Several previous studies support our postulations that full-length membrane α-Klotho is a necessity in modulating EC response to FGF-23. Previously, Saito *et al*. showed that EC function depends on α-Klotho using a mouse α-Klotho knockout model [14]. Furthermore, it has been shown that ECs express FGF receptors and respond to FGF-23 when using a mouse aortic ring model [20, 21]. Likewise, Richter *et al*. utilized coronary artery ECs to highlight that in states of α-Klotho deficiency, FGF-23 mediated NO production is attenuated, leading to an increase of reactive oxygen species (ROS) [22]. Two recent studies also showed that α-Klotho expression in human ECs declined under uremic status and with cell aging, which might account for vascular dysfunction under these conditions [23, 24].

Given the importance of endogenous α-Klotho, it is interesting that our data displayed its absence in ECs of human brain microvessels. Traditionally, ECs of human brain microvessels (the major component of the blood-brain barrier (BBB)) could be distinguished from other tissue ECs due to several unique characteristics, with the most important being the highly resistant tight junctions that provide high transendothelial electrical resistance and retard paracellular flux [25, 26]. We provide a new unique property of HBMECs to explore: lack of endogenous α-Klotho expression despite the presence of FGFR1, FGFR3, and FGFR4. Whether the absence of endogenous α-Klotho expression plays a role in the physiology of brain microvascular tight junction structure and function is unknown. The significance of this absence would also need further investigation. Several recent studies provide clues to the role of Klotho in brain microvessels [27, 28]. Soluble α-Klotho reverses endothelial hyperpermeability observed in the α-Klotho-deficient mouse model [27], resulting from direct binding of α-Klotho to VEGF receptor 2 and the transient receptor potential canonical-1 Ca^2+^ channel and restoring endothelial Ca^2+^ handling. Another cell surface receptor system modulated by hormonal α-Klotho could also be essential for brain microvascular ECs integrity and physiological function. A α-Klotho overexpressing mouse model experienced extended life span that may in part be due to α-Klotho binding to a cell-surface receptors, resulting in repressed intracellular signals of insulin and insulin-like growth factor 1 pathways [28].

ECs are very well placed to respond to humoral α-Klotho protein, particularly on the cell surface exposed to plasma. It is therefore interesting to note that soluble α-Klotho was able to mitigate phosphate and FGF-23 mediated endothelium dependent vascular contractility through increased NO production [21]. Endothelial dysfunction in an α-Klotho-deficient mouse was improved by establishing parabiosis with wild-type mouse [29]; however, with our experimental setup we could not see an effect of soluble α-Klotho in any of the EC models. When ex-vivo artery or ECs were treated with FGF-23, the observed response likely involved locally, endothelial synthesized α-Klotho [20]. EC dysfunction was also rescued in a multiple risk factor syndrome rat model after full-length, transmembrane *in vivo* α-Klotho gene delivery [15]. Liu *et al*. showed the importance of cellular α-Klotho, utilizing HUVECs and human fibroblasts to investigate the role of α-Klotho in cellular inflammatory response to cell senescence. They showed that intracellular, not circulating, α-Klotho was essential for suppressing senescence associated inflammation [30]. These studies suggest that a complex overlap in function between soluble, cleaved, and transmembrane α-Klotho is likely.

Previous clinical studies have shown that elevated circulatory FGF-23 is associated with endothelial dysfunction in CKD patients [31]. Similarly, hyperphosphatemia has been shown to drive the development of accelerated cardiovascular disease in CKD patients [32]. Our study provides evidence for a possible mechanism by showing that high phosphate can suppress endogenous α-Klotho expression leading to mitigation of FGF-23’s effects on eNOS up-regulation in ECs. In CKD, uremic stress leads to a state of endothelial FGF-23 resistance through suppression of α-Klotho expression. In the absence of a functional FGF-23/α-Klotho hormonal system, this leads to failure of endothelial function with resultant pathologic modulation of eNOS expression and vascular homeostasis. Notably, while our results showed that high phosphate significantly decreased the expression of α-Klotho, low levels of α-Klotho protein could still be detected in HAECs; thus the precise mechanism by which hyperphosphatemia functions to mitigate the FGF-23’s effects requires further studies. In contrast with other groups, we did not observe eNOS down-regulation after high phosphate treatment [33]. This may be in part due to our use of HAECs, compared to the more commonly utilized HUVECs.

As stated recently, Richter *et al*., using human coronary artery ECs, also showed that NO production increases after FGF-23 treatment and this was dependent on the presence of α-Klotho [22]; however, their results suggest that these effects are due to the secretion of the soluble α-Klotho. In contrast, our study highlights that the addition of soluble α-Klotho to HAECs following α-Klotho knockdown was unable to rescue EC resistance to FGF-23. This would mean that HAEC responses to FGF-23 are mainly due to the full-length membrane α-Klotho, not soluble α-Klotho. Additionally, their results suggest that increased NO production is due to FGF-23 mediated eNOS activation, whereas we showed increases in both eNOS expression and activation. The differing results might be due to different types of vessels and ECs. Lastly, the authors also found that in states of Klotho deficiency, FGF-23 would promote oxidase stress. Whether FGF-23 also mediates endothelial function via ROS pathway in ECs of different vessels requires further studies.

In addition, our results also showed a dose-dependent response of HAECs to FGF-23, raising an important consideration for future studies into clinical application. The dose of FGF-23 required to stimulate proliferation was much higher than the dose to increase eNOS expression and NO production in HAECs. Mechanisms of vascular injury such as age-related vascular dysfunction usually begin with a lower expression or inactivation of eNOS followed by decreased production of NO [34]. Therefore, a lower dose of FGF-23 might be enough to restore EC function at this earlier stage of disease. At the late stage of vascular injury, when ECs are committed to necrosis or apoptosis [34], a higher dose might be needed for FGF-23 to stimulate EC proliferation and rescue cell loss. An *in vivo* study setup in the future would be needed to validate this postulation.

Our study has some limitations. First, these mechanistic studies only used *in vitro* cellular experiments; our findings would need validation by additional *in vivo* studies. We recognize that the primary HBMECs monolayer culture system may be limited by HBMEC transformation and loss of specific BBB features. While single-cell type culture systems are indispensable to studying the role of individual cell-types, our data will need to be interpreted cautiously against these limitations. It is important to highlight that immunostaining of tissue provided the same observation as the *in vitro* work and both primary endothelial cell cultures were grown in comparable cell culture conditions. Future work using a co-culture systems or organ culture may in part address these concerns and help confirm our data. Furthermore, in this study, we have utilized brain tissue from the cerebellum and hippocampus regions to investigate the expression of α-Klotho in brain microvessels. As previously demonstrated by our group [13], α-Klotho is widely distributed throughout the cerebral cortex and this tissue type has not been included, in part due to limitations of available tissue. A thorough examination of brain tissues would be needed to validate the absence of α-Klotho expression throughout the human brain microvasculature. Lastly, although FGF-23 can produce endothelial cell response through several major pathways including via PI3K/Akt and MAPK pathways [16, 35], we focused on the detection of eNOS and NO synthesis. Consequently, the precise mechanisms by which hyperphosphatemia functions to mitigate the FGF-23's effects, the signaling pathways involved by which FGF-23 increase eNOS and NO production, and other significant endogenous α-Klotho mediated signaling functions will require further studies.

In summary, our results have shown that human ECs differentially express functional full-length α-Klotho protein. And that it is this full-length α-Klotho protein that modulates EC response to FGF-23. We speculate that intracellular, cell surface and cleaved α-Klotho is likely to play different roles for endothelial and vascular function. Future studies will have to investigate its potential role in health and diseases.

## Supporting information

S1 Fig Optimization of α-Klotho knockdown efficacy in human aortic endothelial cells. (TIF)
