# Supplementary figures and images for: α-Klotho expression determines nitric oxide synthesis in response to FGF-23 in human aortic endothelial cells
Source: PLoS One. 2017 May 2;12(5):e0176817. doi: 10.1371/journal.pone.0176817 (PMC5413063; doi:10.1371/journal.pone.0176817)

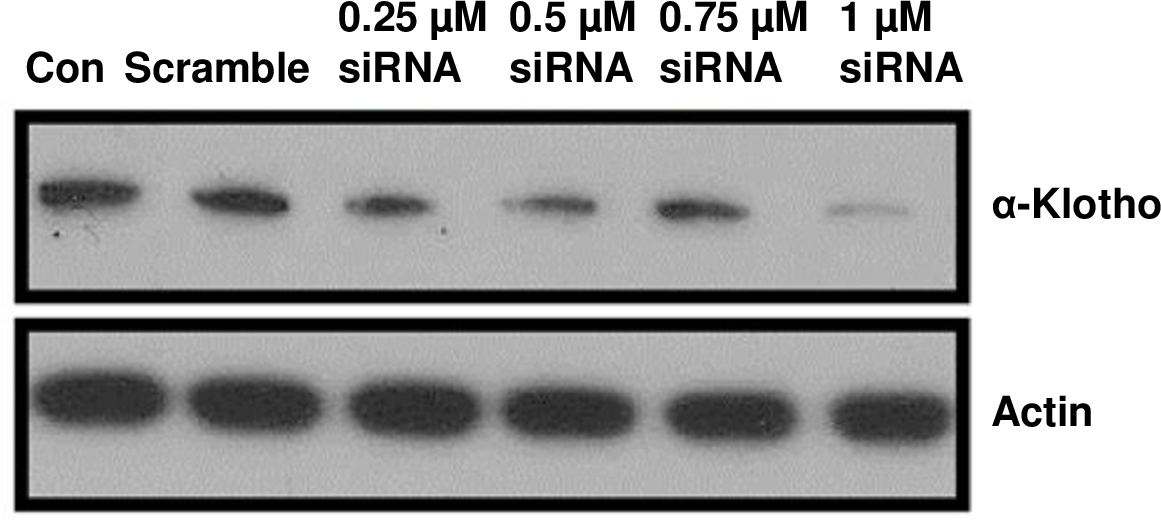

Supplement: S1 Fig — (TIF) [file pone.0176817.s001.tif]
